# Functional Capacity, Lipid Profile, and Associated Factors in Older Adults Living in Urban and Rural Areas

**DOI:** 10.1155/2022/9820221

**Published:** 2022-10-10

**Authors:** Francieli Cristina Comelli Medeiros, Clodoaldo Antônio De Sá, Eduardo Ottobeli Chielle, Fátima Ferretti, Rosana Amora Ascari, Gabriela Peretro, Vanessa da Silva (Corralo)

**Affiliations:** ^1^Universidade Comunitária da Região de Chapecó-Unochapecó, School of Heath, Health Science Post-Graduate Program, 295-D Servidão Anjo da Guarda Street, Chapecó, Santa Catarina, Brazil; ^2^Universidade Do Oeste de Santa Catarina, Department of Life and Health, 211 Oiapoc Street, São Miguel Do Oeste, Santa Catarina, Brazil; ^3^Universidade Do Estado de Santa Catarina, Department of Nursing, 680E Beloni Trombeta Zanin Street, Chapecó, Santa Catarina, Brazil

## Abstract

This study aimed to analyze the relationship between sociodemographic and lifestyle variables, functional strength, aerobic capacity, and lipid profile of older adults living in urban and rural areas. For this purpose, 208 older adults were evaluated (132 living in rural areas and 73 living in urban areas). Sociodemographic data were collected, and functional strength, aerobic capacity, and lipid profile of older adults living in the southern region of Brazil were evaluated. Only total cholesterol and LDL cholesterol were associated with place of residence (*p* < 0.05), and living in the countryside was associated with low aerobic capacity (*p*=0.010). The use of medication (OR = 3.01; *p*=0.012) and smoking (OR = 0.30; *p*=0.027) were the only variables that explained aerobic performance, regardless of place of residence. In conclusion, place of residence is not a factor that, alone or in combination with other conditions, affects the functional performance or lipid profile of the older adult population.

## 1. Introduction

Population aging is a global and multidimensional phenomenon that takes place in a close relationship with the environment in which older adults are inserted, involving factors such as family and community life, culture, lifestyle, and economic condition. As a natural consequence of this phenomenon, there is a concomitant increase in the incidence of chronic diseases and length of time living with diseases such as diabetes, hypertension, dementia, and dyslipidemia [1]. In this context, several studies around the world have focused on rural populations. These studies have mainly assessed aspects such as falls [2], psychological aspects [3], hypertension [4], functional capacity [5] dyslipidemias [6], and quality of life [7,8].

The relationship between changes in the lipid profile and risk of mortality from cardiovascular diseases is well established in the scientific literature. An active lifestyle shown to be closely related to lower mortality from cardiovascular diseases and physical activity or exercise significantly reduces the overall risk for all cardiovascular diseases [9].

In the literature to which we had access, we did not find studies assessing the profile of older adults according to their place of residence and taking aspects into consideration such as functional strength and aerobic capacity. Considering that functional strength is associated with greater autonomy in activities of daily living (ADL) and that aerobic capacity is a good indicator of general physical fitness, assessing how these factors affect the lipid profile depending on the place of residence will allow us to identify in which dimensions policies and actions aimed at promoting the health of older adults living in rural or urban areas should be similar or different.

In his doctoral thesis, Mattos [10] assessed rural and urban older adults in the state of São Paulo and highlighted that the practice of high-intensity physical activity was more prevalent in the former (72.7%), while moderate or low-intensity physical activity prevailed among urban older adults (36.2 and 11.2%, respectively). In this study, dyslipidemia was more prevalent among older adults living in rural areas. Zhou et al. [6] also showed higher prevalence of dyslipidemia in the rural population. However, functional assessments were not performed in these studies, and older adults were not included in one of them.

Several factors that influence the health of older adults may be aggravated by the place of residence. For example, access to healthcare services is less accessible to the rural population due to the distance, poor conditions of the road or absence thereof, poor transportation, and so on [11].

In view of the abovementioned aspects, rural and urban older adults may have differences in the lipid profile. Nevertheless, several social and economic factors or even factors related to the level of physical fitness may be more relevant for the lipid profile than the place of residence itself. These multiple factors have unique interactions in different countries or different regions of the same country. Thus, determining to what extent factors associated with the place of residence (e.g., lifestyle, sociodemographic characteristics, and functional performance) influence the lipid profile of older adults is essential to plan strategic actions and public policies with the potential to positively affect the lipid profile and, consequently, the risk for the development of cardiovascular diseases.

Population aging is a global reality and one of the greatest current public health challenges, especially when it comes to the identification of potential conditions for the prevention of chronic noncommunicable diseases or reduction of associated problems in the older adult population. Sociodemographic factors and lifestyle may affect older adults who reside in urban and rural environments differently. In this sense, this study aimed to analyze the relationships between sociodemographic and lifestyle variables, functional strength, aerobic capacity, and lipid profile of urban and rural older adults.

## 2. Method

A cross-sectional descriptive study was conducted in the period from November 2019 to March 2020, where older adults living in the city of São Miguel do Oeste, located in the far western region of the state of Santa Catarina, Brazil, were assessed.

### 2.1. Study Participants

We randomly selected 205 older adults from 13 urban neighborhoods (*N* = 132) and seven rural locations or rural settlements in the city (*N* = 73). The sample was equalized by place of residence, sex, and age group (60 to 79 years and 80 years or more). Participants who did not obtain the minimum score in the Mini-Mental State Examination (MMSE) for their level of education [12,13] and those who were not found at home in up to three visits on nonconsecutive days were excluded from the study.

### 2.2. Procedures

#### 2.2.1. Cognitive Assessment

The Mini-Mental State Examination (MMSE) was used to assess global cognitive functioning. This test investigates orientation to time and space, attention, language, constructive skills, and immediate/delayed memory [12,14]. The cutoff points suggested by Bertolucci et al. [13] were used (13 points for illiterates, 18 points for individuals with low or medium level of education, and 26 points for individuals with high level of education). Older adults who did not obtain the minimum scores (adjusted for education level) were not included in the sample.

#### 2.2.2. Sociodemographic Aspects and Lifestyle

For the collection of sociodemographic and lifestyle data, an interview was carried out using a questionnaire adapted from the study by Morais et al. [15]. Sections 1, 3–5 of the instrument were used, addressing the following aspects:

- Personal information: sex, skin color, place of birth, length of residence in the urban and/or rural area, marital status, schooling, religion, retirement, retirement amount, economic assistance from third parties, and previous occupation
- Housing conditions: type of construction, piped water, water source, sewage, existence of bathrooms, existence of electricity, garbage collection, existence of household goods, greater expenditure of money in the last 6 months, means of communication used, and means of transport used by older adults
- Family composition: assessed whether the research participant lived alone or with a companion and detailed who the companions were (spouse, child, caregivers, etc.).
- Health conditions and lifestyle: self-perception of health, comparison with the health of other people of the same age, use of medication, weekly physical activity, smoking habits, number of meals per day, use of alcohol, type of diet, hydration, hearing problems, vision problems, presence of teeth, presence of dental prosthesis, number of falls in the last year, fracture sites, and self-reported health problems

#### 2.2.3. Assessment of Functional Strength and Aerobic Capacity

The chair stand test [16] and the two-minute step test [17] were used to assess the functional strength and aerobic capacity, respectively.

#### 2.2.4. Collection and Analysis of Blood Samples

All blood samples were collected at homes of the participants on previously scheduled dates and times. The participants were informed about the necessary requirements for the collection of blood samples on the scheduled date. On the day before collection, each participant was contacted again to confirm the collection and review the procedures. The samples were collected after a fasting period of 10 to 12 hours. A new date was scheduled in the case of individuals who were not fasting at the time of collection.

Nine milliliters of blood were collected from each participant in a vacuum system with a clot activator. The entire blood collection procedure was performed by trained and qualified professionals, thus respecting all biosafety procedures recommended for this purpose. Immediately after collection, all samples were properly stored and sent to the Biochemical Chemistry Laboratory of the Western University of Santa Catarina, São Miguel do Oeste Campus. A biochemical analyzer was used for the analysis of the lipid profile (total cholesterol, triglycerides, HDL cholesterol, and LDL cholesterol) (Cobas C111, Roche Diagnostics™, Switzerland).

### 2.3. Data Collection

All data collection was performed at homes of the participants in the period from November 2019 to March 2020 (data collection was stopped due to the COVID-19 pandemic). After the initial contact made by community health workers, home visits were scheduled. In this first visit, the researcher explained all procedures of data collection and the objectives of the study. The older adults who agreed to participate in the research were assessed by the Mini-Mental State Examination, and when they obtained the minimum score according to their level of education, they signed the informed consent form, agreeing to participate in the research. After that, each participant filled out a form addressing sociodemographic data and family composition, housing conditions, and lifestyle. Then, they performed the chair-stand test and, 10 minutes later, the two-minute step test. The collection of blood samples was scheduled for a later date, considering the logistics of collection at the participant's home, which aimed at the collection of the largest number of variables possible at the same time and viability of the samples, considering the adequate storage time before the analyses.

### 2.4. Ethical Aspects

This study was conducted in accordance with the recommendations of resolution number 466/2012 of the National Health Council, which establishes the guidelines and regulatory standards for research involving human beings. This project was approved by the Human Research Ethics Committee (protocol number 3,624,101).

### 2.5. Data Analysis

A double-entry spreadsheet database was built using the Microsoft Excel® program. Descriptive statistics were used to present the results through absolute (*n*) and relative (%) frequencies. The analyses of associations between the variables of the functional tests and the lipid profile were performed using the chi-square test (*χ*^2^). Logistic regression was used to estimate the influence of variables on the behavior of functional tests and biochemical factors. The statistical procedures were performed using STATA (version 16.0), adopting a significance level of *p* ≤ 0.050.

## 3. Results

The sample consisted of 205 older adults, predominantly living in the urban area (64.4%). The sociodemographic characteristics, housing conditions, health conditions, and lifestyle aspects of the participants are shown in Table 1.

The performance in the chair stand test and the two-minute step test (Table 2) showed that only aerobic capacity had a statistically significant association with place of residence (*x*^2^ = 9.25; df = 2; *p*=0.010). Regarding the lipid profile (Table 3), only total cholesterol (*x*^2^ = 6.52; df = 2; *p*=0.038) and LDL cholesterol (*x*^2^ = 9.35; df = 3; *p*=0.025) showed a statistically significant association with place of residence.

The logistic regression (Table 4) showed that changes in total cholesterol were explained by sex (OR = 1.99; *p*=0.032), while changes in LDL cholesterol were explained by the number of full meals (OR = 2.75; *p*=0.026). The other analyzed factors (housing, age group, skin color, marital status, medication use, smoking, alcoholism, physical activity, fluid intake, hearing problems, vision problems, dental problems, falls, and performance in the chair stand test and the two-minute step test) did not show statistical significance to be included in the model (*p* > 0.05).

The analysis of the factors that influenced the performance in the chair stand test and two-minute step test, only medication use, and smoking were identified as factors significantly affecting (*p* < 0.05) the performance in the two-minute step test [17]. None of the analyzed factors showed statistical significance to be included in the regression model to explain the performance in the chair stand test (*p* > 0.05).

## 4. Discussion

### 4.1. Profile of Participants According to the place of Residence

Of the older adults who participated in this study, most were female (urban = 67.4%, rural = 56.2%), aged between 60 and 69 years (urban = 78.0%, rural = 89.0%), and declared to be literate (urban = 94.0%, rural = 90.4%). All residences visited had piped water and electricity, and 87.9% of urban older adults and 91.8% of the ones from rural areas lived in their own homes. Only 3% of rural and 5% of urban older adults were not retired or pensioners.

The higher number of women in our study in relation to men can be explained by longer life expectancy of women resulting from their lower exposure to risk factors and their adherence to health care, as well as to the existence of health services and preventive and therapeutic programs aimed specifically at women. Practically all over the world, women live longer than men do. This is a phenomenon called the feminization of aging. This advantage of women over men can partially be attributed to the differences in diseases that affect these two groups. The rate of lethal diseases has shown to be higher among men. Nonfatal but disabling diseases and chronic diseases, including arthritis and hypertension predominate among women, while ischemic heart disease prevails in men. Older women have shown higher morbidity rates than older men, but mortality rates are lower in women than in men for the same diseases [18].

As for working conditions, only 13.5% of the older adults from urban areas were still working, while in rural areas, 53.4% were still engaged in their work activities. The profile of rural properties in the far western region of Santa Catarina, characterized by small family farms based on the cultivation of tobacco, corn, fruit crops, and dairy products [19], is a determining factor for older adults to continue working in the field, even after retirement.

Living alone was not common among urban and rural older adults (22.7% and 5.5%, respectively); 66.7% of older adults from urban areas and 43.5% of those from rural areas lived with their spouses. The participants from both the rural and urban areas believed that their health was good or excellent (58.3% and 60.3%, respectively). Conversely, comparative health was rated as good or excellent by more than 70% of older adults (urban = 74.2%, rural = 76.7%). Among the study participants, more than 82% used medications (urban = 78.9% and rural = 82.2%).

Both urban and rural older adults reported relatively high alcohol consumption, especially the latter (urban = 40.9%, rural = 53.4%). Conversely, eating three full meals and drinking more than five glasses of water per day prevailed in both urban (85.6% and 70.5%, respectively) and rural (82.2% and 80.8%, respectively) settings.

Studies show that the greater the exposure to smoking, the worse the physical health. Also, higher levels of nicotine dependence are associated with lower quality of life scores, higher levels of disability, and lower life expectancy [20,21]. In this study, smoking was reported by 17.8% of the older adults living in rural areas and only 3% of those living in urban areas. These data indicate the need for actions to combat smoking, especially among older adults living in rural areas.

Problems related to vision and dentition were the most prevalent (vision problems: urban = 87.1%, rural = 76.7%; total absence of teeth: urban = 56.1%, rural = 82.2%). Hearing problems were reported by 18% and 49.3% of the older adults living in rural and urban areas, respectively.

Although the number of falls reported by the older adults in the last 12 months was low (urban = 21.2%, rural = 10.1%), it is important to note that the lack of physical exercise (reported as “no practice of physical exercise”) was quite high, especially in the rural population (urban = 56.1%, rural = 82.2%). On the other hand, some studies, such as Ferretti et al. [22], state that rural older adults remain more active than urban older adults because physical activities performed by these two groups can differ in the type and intensity of activity. In rural environments, occupational activities prevail, predominantly agricultural work (family and extensive or subsistence agriculture), domestic activities, livestock rearing, plant extraction, beekeeping, among others. In turn, the activities practiced by older adults in urban areas are more related to the use of technologies (e.g., cell phones and TV), with a consequent tendency to lower intensity of work and domestic activities.

Although this study did not assess the level of physical activity using the International Physical Activity Questionnaire (IPAQ), our data showed, in general, a predominance of older adults with low performance in functional tests. Low aerobic performance was predominant among rural older adults: 74% of them did not practice any kind of physical activity. Ribeiro et al. [7] found a different result, with a predominance of active older adults in rural areas, while insufficiently active or sedentary individuals prevailed in urban areas. This contrast reflects a set of characteristics peculiar to each region. Although the study by Ribeiro et al. [7] also included older adults from the Palmas region of the state of Paraná in Southern Brazil, the characteristics of rural areas differ from those of the present study. In another study with rural and urban older adults in the city of Florianópolis, Santa Catarina, the former remained in work for a longer time than the latter [23]. In a study conducted in Peru, Hernandez-Huayta et al. [24] demonstrated that most of the older population of rural areas still works, which is associated, in this case, with the need to maintain income and the short time analyzed in the study.

### 4.2. Functional Strength and Aerobic Capacity

One of the main findings of the present study was the fact that the place of residence was not a determining factor for performance in tests of functional strength and aerobic capacity. In the chair stand test, 66.8% of the older adults had low functional strength performance, although the prevalence of this condition was not associated with the place of residence. Low performance in the two-minute step test was more prevalent in rural than that in urban areas (rural = 89%, urban = 74%). Low performance in the two-minute step test reflected the practice of physical exercises in the rural older adult population, since 74% of them said that they did not practice any type of physical exercise.

A sedentary lifestyle is a factor that increases the risk of developing chronic diseases, including hypertension. It is known that a sedentary lifestyle is related to a reduction in maximal aerobic capacity, muscle strength, motor responses, and overall functional capacity [25], which is compatible with reduced ability to perform ADLs. The fact that, in this study, 57.5% and 72% of the rural and urban older adults, respectively, showed low performance in functional strength is worrisome, considering the relationship between low functional performance and falls in this population.

The regression analysis showed that the place of residence, sex, and all sociodemographic or lifestyle factors analyzed in this study did not predict functional strength performance. This reinforces the importance of regionalized studies, since the set of factors that potentially affect functional strength performance may differ considerably in different contexts.

Although low performance in the two-minute step test was more prevalent among rural older adults, the place of residence was not a predictor of aerobic performance. The only predictors of aerobic capacity were the use of medications and smoking. In this study, the use of medications decreased the aerobic performance of older adults by three times in relation to nonconsumption of medicines. This result may be explained by the fact that the use of medications is associated with a greater number of comorbidities and chronic diseases, conditions often associated with worse general physical fitness. Conversely, the practice of smoking was associated with a 70% reduction in aerobic capacity. The deleterious effects of smoking on aerobic fitness are well established by science; however, it should be noted that smoking is also an independent risk factor for cognitive decline [26] and has important deleterious effects on the cardiac structure and function [27].

In the study by Silva et al. [28], the level of physical activity was inversely associated with medication use. A Brazilian population-based study by Bertoldi [29] with 3,182 individuals who answered the IPAQ-short version and self-reported consumption of medications found that sedentary individuals consumed 23% more medications than more active individuals. These data differ from those used in this study. We found that urban older adults reported using more medications and showed higher adherence to weekly physical exercises (62.1%), while only 26% rural older adults reported practicing some type of physical exercise. Lower practice of physical exercise in the rural population may be related to factors such as less access to exercise centers such as gyms or groups of older adults who come together for this purpose and the large distance among properties which make social gatherings and even access to residences difficult.

#### 4.2.1. Lipid Profile of Rural and Urban Older Adults

Among the lipid profile, only total cholesterol and LDL cholesterol were associated with place of residence. Desirable or optimal total cholesterol and LDL cholesterol values were more prevalent in urban than in rural older adults (total cholesterol: rural = 60.3%, urban = 74.2%; HDL cholesterol: rural = 60.3%, urban = 72.7%).

Among the studied variables, only sex explained the behavior of total cholesterol according to the regression analysis. In this study, being male increased the risk of having altered total cholesterol by two times regardless of place of residence. In turn, LDL cholesterol concentrations were explained only by the number of full meals the older adults had per day, regardless of sex and place of residence. Each additional full meal taken per day (two or three) increased the chance that the surveyed older adults had altered LDL cholesterol levels by 2.7.

Although the adequate distribution of meals is important, the quality of food, observing the appropriate proportions of macronutrients and micronutrients, is a determining factor for the prevention and control of dyslipidemia. An adequate diet is one of the first-choice treatments for dyslipidemia and other metabolic diseases because it promotes the reduction of weight and visceral fat, thus directly affecting the lipid profile. A study by Jacondino et al. [30] showed that 66.1% of the surveyed older adults were classified as nonadherent to lifestyle modification proposals, especially regarding dietary practices. According to these authors, the main reasons for nonadherence to the diet were: “lack of persistence” (38.9%) and “perceived context” [17]. This last justification was related to the palatable aspects of food; that is, the older adults do not think that whole foods are tasty, opting for white flour foods, preferring sugar over the sweetener, and consuming more salt than recommended. They said that they do not find food appetizing if these elements are absent or reduced. The lack of persistence was also justified by other reasons (15.3%), including lack of financial resources or because they considered the measure unnecessary. In addition, psychological factors may be relevant in eating behaviors, particularly among individuals diagnosed with depression, since the presence of some manifestations of this mood disorder, such as appetite distortion, feelings of worthlessness, low self-esteem, fatigue, and loss of energy and motivation, may affect treatment adherence.

Dyslipidemia is a risk factor for noncommunicable diseases (NCDs), including cardiovascular disorders, which are responsible for 35% of the deaths in older adults aged 80 years or more and around 32% among older adults aged between 60 and 79 years [31]. In this sense, dietary education strategies, including food choices, preservation, and preparation forms, among others, should be implemented among the older adult population to enhance the effects of good eating habits on the lipid profile.

In this study, low performance in the two-minute step test and low prevalence of the practice of physical exercise, especially among older adults in rural areas, constitute a risk factor for dyslipidemia. They are, consequently, important risk factors for cardiovascular diseases. In this context, it is important to underline the importance of actions to promote the practice of physical exercise, especially among older adults living in rural areas, considering that the regular practice of physical exercise combined with adequate nutrition improves the lipid profile and reduces the risk of development of chronic diseases, especially cardiovascular diseases.

In general, our data highlighted that the promotion of the regular practice of physical exercise among older adults regardless of their place of residence is urgently needed, and although eating habits were not within the scope of this research, there is important evidence pointing to the need to include nutritional education strategies for this population.

## 5. Conclusion

The analysis of the collected data allows us to conclude that regardless of the place of residence (urban or rural), the use of medication worsens aerobic performance by three times, while smoking reduces aerobic capacity by 70%. Each full meal per day increases the chance of having increased LDL cholesterol levels by 2.7 times among older adults.

Therefore, the place of residence is not a factor that, alone or in combination with other conditions, affects the functional performance or the lipid profile of the older adult population.

## Figures and Tables

**Table 1:** Sociodemographic, housing, and lifestyle characteristics of older adults living in São Miguel do Oeste, SC, Brazil, 2020 (*n* = 205).

Variables	Categories	Urban		Rural	
		*n*	%	*N*	%
Gender	Female	89	67.4	41	56.2
	Male	43	32.6	32	43.8

Age group	60–79 years	103	78.0	65	89.0
	80 years or more	29	22.0	8	11.0

Skin color	White	123	93.2	73	100.0
	Brown	8	6.1	0	0.00
	Black	1	0.5	0	0.00

Marital status	Married	74	56.1	50	68.5
	Widowed	47	35.6	14	19.2
	Single	3	2.3	7	9.6
	Divorced	5	3.8	1	1.4
	Other	2	1.5	1	1.4
	Separated	1	0.8	0	0.0

Can read and write	Yes	124	94.0	66	90.4
	No	4	3	4	5.5
	Just sign the name	4	3.0	3	4.1

Household	Own	116	87.9	67	91.8
	Rented	10	7.6	1	1.4
	Assigned	5	3.8	3	4.1
	Others	1	0.8	2	2.7

Running water	Yes, inside the house	132	100.0	73	100.0

Energy	Yes	132	100.0	73	100.0

Retirement	Yes, due to age	48	36.4	48	65.8
	Yes, due to length of service	51	38.6	10	13.7
	Yes, plus pension	16	12.1	5	6.8
	Yes, due to disability	9	6.8	4	5.5
	No	4	3.0	4	5.5
	Pensioner	4	3.0	2	2.7

Has other income	No	98	74.2	50	68.5

Receives financial help	No	114	86.4	66	90.4

Currently working	No	113	85.6	34	46.6

Occupation	Farming	53	40.2	63	86.3
	Housewife/domestic	26	19.7	2	2.7
	Teacher	8	6.1	1	1.4
	Merchant	8	6.1	0	0.00
	Others/self-employed	37	28.1	7	9.6

Lives	Accompanied	102	77.3	69	94.5

Lives with	Spouse	68	66.7	30	43.5
	Children/sons-in-law/grandchildren	33	32.5	30	43.5
	Others	1	1.0	9	13.0

Self-perceived health	Excellent	15	11.4	11	15.1
	Good	62	47.0	33	45.2
	Regular	45	34.1	26	35.6
	Bad	9	6.8	1	1.4
	Lousy	1	0.8	2	2.7

Comparative health	Excellent	26	19.7	22	30.1
	Good	72	54.5	34	46.6
	Regular	30	22.7	14	19.2
	Bad	4	3.0	1	1.4
	Lousy	0	0.00	2	2.7

Medication use	Yes	116	87.9	60	82.2

Smoking	No	128	97.0	60	82.2

Alcoholism	Do not know	78	59.1	34	46.6
	Drink eventually	44	33.3	31	42.5
	Drink up to 3 times a week	1	0.8	2	2.7
	Drink 3 to 7 times a week	5	8.0	6	8.2
	Already drank in the past	4	3.0	0	0.00

Full meals	One	7	5.3	1	1.4
	Two	12	9.1	12	16.4
	Three	113	85.6	60	82.2

Fluid intake	Less than 3 glasses	9	6.8	0	0.00
	From 3 to 5 glasses	30	22.7	14	19.2
	More than 5 glasses	93	70.5	59	80.8

Hearing problems	No	95	72.0	37	50.7

Vision problems	Yes	115	87.1	56	76.7

Dental problems	No teeth	74	56.1	60	82.2
	Yes, only in the lower part of the mouth	22	16.7	19	26.0
	Yes, only in the upper part of the mouth	7	5.3	0	0.00
	Yes, less than half the teeth	20	15.2	14	19.2
	All the teeth	9	6.8	3	4.1

Physical activities	None	50	37.9	54	74.0
	Walk	53	40.2	14	19.2
	Others	29	22.0	5	6.9

Falls	No	104	78.8	62	84.9

**Table 2:** Distribution of the scores of the chair stand test and the two-minute stationary walk test according to the place of residence of older adults in São Miguel do Oeste, SC, Brazil, 2020 (*n* = 205).

		Place of residence				Total	
		Rural		Urban			
		*N*	%	*n*	%	*N*	%
Chair stand test (*x*^2^ = 4.44; gl = 2; *p*=0.108)	High	1	1.4	1	0,8	2	1,0
	Medium	30	41.1	36	27.3	66	32.2
	Low	42	57.5	95	72.0	137	66.8

Stationary walk test (*x*^2^ = 9.25; gl = 2; *p*=0.010)	High	1	1.4	0	0.0	1	0.5
	Medium	7	9.6	34	25.8	41	20.0
	Low	65	89.0	98	74.2	163	79.5

Total		73	100.0	132	100.0	205	100.0

**Table 3:** Distribution of blood glucose, cholesterol, triglycerides, HDL, and LDL classification according to the place of residence of older adults (*n* = 205).

		Place of residence				Total	
		Rural		Urban			
		*n*	%	*n*	%	*n*	%
Total cholesterol (*x*^2^ = 6.52; gl = 2; *p*=0.038^*∗*^)	High	7	9.6	14	10.6	21	10.2
	Altered	22	30.1	20	15.2	42	20.5
	Ideal	44	60.3	98	74.2	142	69.3

Triglycerides (*x*^2^ = 1.77; gl = 2; *p*=0.412)	High	6	8.2	19	14.4	25	12.2
	Maximum	10	13.7	15	11.4	25	12.2
	Normal	57	78.1	98	74.2	155	75.6

HDL cholesterol (*x*^2^ = 4.66; gl = 2; *p*=0.093)	Low	30	41.1	53	40.2	83	40.5
	Normal	43	58.9	71	53.8	114	55.6
	Excellent	0	0.0	8	6.1	8	3.9

LDL cholesterol (*x*^2^ = 9.35; gl = 3; *p*=0.025^*∗*^)	High	13	17.8	15	11.4	28	13.7
	Maximum	16	21.9	21	15.9	37	18.0
	Desirable	26	35.6	35	26.5	61	29.8
	Excellent	18	24.7	61	46.2	79	38.5

Total		73	100.0	132	100.0	205	100.0

**Table 4:** Summary with the independent variables included in the logistic regression models to explain the behaviors of the lipid profile and blood glucose variables of older adults.

Independent variables	Dependent variables					
	Total cholesterol		LDL		Stationary walk test	
	OR	*p*	OR	*p*	OR	*p*
Gender	1.99	0.032	—	—	—	—
Medication use	—	—	—	—	3.01	0.012
Smoking	—	—	—	—	0.30	0.027
Full meals	—	—	2.75	0.026	—	—

OR: odds ratio.

## Data Availability

Data from the present study can be made available upon request from the corresponding author

## Additional Points

This study adds the following to the existing knowledge: most of the studies comparing health and physical fitness aspects of rural and urban older adults compare the rural population with large urban centers. This study showed that the place of residence is not a determining factor to predict the motor performance and lipid profile of older adults when evaluating small countryside cities. Regardless of the place of residence (urban or rural), (i) the use of medication worsens aerobic performance by three times and smoking reduces aerobic capacity by 70%; (ii) each full meal per day increases the chance of older adults having increased LDL cholesterol levels by 2.7 times. What are the key implications for public health interventions, practice, or policy? (i) There are no effective strategies for preventing dyslipidemia and promoting physical exercise among older adults regardless of place of residence. (ii) Health promotion and disease prevention actions must be based on data that take into account knowledge about the local reality. (iii) Data from studies that assess locoregional characteristics are critical to the design of global health promotion policies for the older adult population.
